# The magnitude of antibiotic resistance to *Helicobacter pylori* in Africa and identified mutations which confer resistance to antibiotics: systematic review and meta-analysis

**DOI:** 10.1186/s12879-018-3099-4

**Published:** 2018-04-24

**Authors:** Hyasinta Jaka, Jee Ah. Rhee, Linda Östlundh, Luke Smart, Robert Peck, Andreas Mueller, Christa Kasang, Stephen E. Mshana

**Affiliations:** 1 Gastroenterology and Hepatology Unit, Weill Bugando School of Medicine, Catholic University of Heath and Allied Sciences and Bugando Hospital Mwanza, P.O. BOX 1464, Mwanza, Tanzania; 2 Weill Cornell Medicine-Qatar, Doha, Qatar; 3 National Medical Library, United Arab Emirates University, Abu Dhabi, United Arab Emirates; 4 Cincinnati Children’s Hospital Medical Centre, Cincinnati, Ohio USA; 5 Center for Global Health, Weill Cornell Medicine, New York, NY USA; 6 Medical Mission Institute Wuerzburg, Wuerzburg, Germany; 7 Department of Microbiology and Immunology, Weill Bugando School of Medicine, Catholic University of Heath and Allied Sciences Mwanza, Mwanza, Tanzania

**Keywords:** *H.Pylori*, Drug resistance, Antibiotics resistance, Clarithromycin, Metronidazole, Quinolones, Africa

## Abstract

**Background:**

Worldwide *Helicobacter pylori* (*H.pylori)* treatment is of great challenge due to increased antibiotic resistance. The burden of *H. pylori* antibiotic resistance in Africa is high with unclear information regarding the real magnitude. This systematic review and meta-analysis was conducted to investigate the magnitude of *H.pylori* antibiotic resistance in Africa to gain insight of the extent of the problem among *H.pylori* naïve treatment patients.

**Method:**

The search was performed in the academic databases, Embase, PubMed, Web of Science and Africa Wide Information. ProQuest Dissertation and Theses, Scopus, Ethos, Africa Index Medicus (WHO), BioMed Central Proceedings, BASE, British Library, Open grey, Library of Congress and the New York Academy of Grey Literature Report were additionally searched for grey literature**.** Published articles from Africa on *H.pylori* antibiotic resistance between 1986 and June 2017 were systematically reviewed to estimate the *H. pylori* extent of resistance to macrolides, quinolones, amoxicillin, tetracycline and metronidazole.

**Results:**

In 26 articles a total of 2085 isolates were tested for metronidazole, 1530 for amoxicillin, 1277 for tetracycline, 1752 for clarithromycin and 823 for quinolones.The overall pooled proportion of *H.pylori* resistance to quinolones, clarithromycin, tetracycline, metronidazole and amoxicillin were: (17.4%, 95%CI 12.8 - 21.9), (29.2%, 95%CI:26.7-31.8), (48.7%, 95%CI: 44.5-52.9), (75.8%, 95% CI: 74.1-.77.4) and (72.6%, 95% CI: 68.6-76.6), respectively. The commonest mutation detected were A2143G (49/97) for clarithromycin, RdxA (41/56) for metronidazole and D87I (16/40) for quinolones.

**Conclusion:**

Prevalence of metronidazole, clarithromycin, and amoxicillin resistance is high in developing world including Africa. This could impair the first line triple therapy of the *H.pylori* infection. There is a need of conducting surveillance of *H.pylori* susceptibility pattern in Africa for dual and triple resistance which can be used for the empirical treatment.

**Electronic supplementary material:**

The online version of this article (10.1186/s12879-018-3099-4) contains supplementary material, which is available to authorized users.

## Background

The prevalence of *H.pylori* infection varies worldwide among different geographical regions and correlates with diverse socio-demographic factors [1]. The *H.pylori* sero-positivity rate is much higher in developing countries than in industrialized countries [2]. The *H.pylori* sero-prevalence in East Africa has been found to range from 54.8% to 75%, with prevalence of 45.7% to 65% reported in Tanzania [3–7]. *H. pylori* antibiotic resistance is increasing worldwide and is associated with low eradication rate [8]. The prevalence of *H.pylori* drug resistance varies from place to place, and is largely depends on the quantity of antibiotics used in that particular population [9, 10]. Overall *H.pylori* antibiotic resistance is of increase in America, Asia and Africa [8, 10].

In developing countries including Tanzania, the first line treatment option against *H.pylori* includes; clarithromycin, metronidazole/ tinidazole, amoxicillin and tetracycline while the second line option includes; levofloxacin, rifabutin and furazolidon. According to the guidelines, the first regimen are the triple-therapy, quadruple therapy and quinolone containing regimen. Triple-therapy treatment regimens consist of proton pump inhibitor and two antibiotics: amoxicillin and clarithromycin, or metronidazole and clarithromycin while quadruple therapy consist of proton pump inhibitor, bismuth and two antibiotics: amoxicillin plus clarithromycin, or metronidazole plus tetracycline. There is Non bismuth quadruple therapy (concomitant) which includes proton pump inhibitor, amoxicillin, metronidazole, clarithromycin or (sequential) five days proton pump inhibitor and amoxicillin followed by seven days proton pump inhibitor, metronidazole and clarithromycin. The second line (salvage therapy) treatment includes quinolone containing regimen which consist of proton pump inhibitor, amoxicillin and levofloxacin and Rifabutin, amoxicillin and proton pump inhibitor [11–14].

The rates of resistance to metronidazole and clarithromycin are increasing worldwide [15, 16]. The metronidazole resistance rate increased from 26.7% in 2010 to 47.2% in 2015, while tetracycline resistance increased from 5.9% in 2010 to 11.7% in 2015 [8, 15]. In addition, the resistance to clarithromycin has been found to increase from 17.2% in 2010 to more than 19.7% in 2017 [8, 15, 17]. In Africa, *H.pylori* treatment is much compromised by antibiotic resistance in about 58%-95% of patients who are treated by the triple therapy [6, 18]. Data on the overall *H.pylori* drug resistance in Africa has never been compiled to show the magnitude of the problem. Therefore, this systematic review and meta-analysis aimed at quantifying the magnitude of *H.pylori* drug resistance in Africa among *H. pylori* naïve treatment patients.

## Methods

### Eligibility criteria

The review included; full articles which investigated on *H.pylori* resistance in any African country among naïve to *H. pylori* treatment patients.

### Information sources

A comprehensive systematic search for literature was conducted in major medical and health science databases, and sources of grey literature. The search was performed in the academic databases, PubMed, Embase, Web of Science and Africa Wide Information. ProQuest Dissertation and Theses, Ethos, Scopus, Africa Index Medicus (WHO), Open Grey, BASE, BioMed Central Proceedings, British Library, Library of Congress and the New York Academy of Grey Literature Report were additionally searched for grey literature.

### Search strategy

Pre-search was conducted between November 2015 and May 2016 to identify relevant keywords and information sources for best possible retrieval of information about *H.pylori* drug resistance in Africa (Additional file 1). A search strategy was initially developed in PubMed and later applied in all selected databases. PubMed’s MeSH was used for systematic inclusion and exclusion of all search terms. This was specifically helpful in identifying all African countries. A list of all previous country names, in addition to current names, compiled based on MeSH “Entry Terms” for each country, and reviewed to only included names from 1986 (Additional file 2).

All keywords included were searched with a combination of the fields “Title”, “Abstract” (alternatively “Topic”) and “Thesaurus”/“Subject Headings”, when available, to maximize the search outcome. No filters or limitations were applied to ensure inclusion of pre-indexed materials. A full search log, including notes and results for all search sessions, can be found in Additional file 2.

### Study selection

**Fig. 1:**
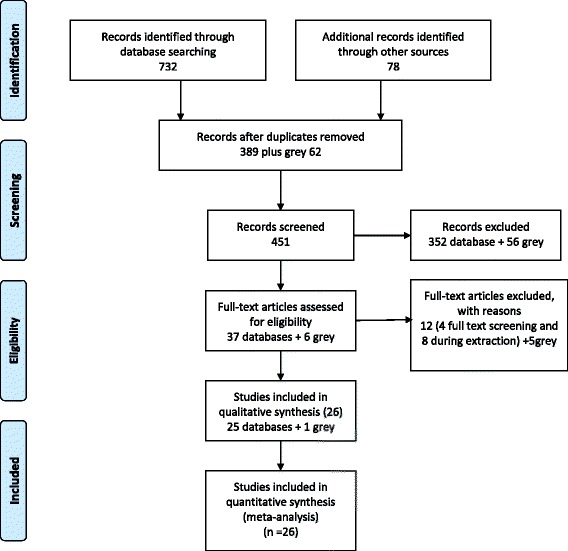
PRSIMA Flow chart

The systematic review software Covidence was used and manual screening was also conducted of all references in the grey literature. Thus, after duplicate removal, a total of 389 abstracts were identified, among these, 352 were excluded due to the following reasons; not about *H.pylori* (213), not about *H.pylori* resistance (86), review and report (25), not Africa (20), conference (2), animal study (1), duplicate (4), guidelines (1). The articles from grey search were 62, among these 56 were excluded and 6 articles were included in the full test assessment. Full-text articles were assessed for eligibility (37 from data base and 6 from grey literature). Out of 43 articles, 17 (12 from data base and 5 from grey literature) were excluded because they had missing information /data, therefore only 26 articles were included in the final analysis (Fig. 1).

### Data collection process and assessment of methodological quality

Relevant articles were independently reviewed by two authors. Data were extracted from each article into an excel spread sheet, which included name of authors, year of publication, country, study population, sample size, samples of isolates, antibiotic tested for resistance, susceptibility results and criteria used and possible mutations detected.

Studies were objectively assessed using the previously validated Newcastle-Ottawa score, which scores of a 6-point scale according to performance in first two categories of sample selection criteria(4) and comparability on the bases of design or analysis(2) while the outcome was not assessed (this is for case control and cohort studies).

### Summary measures

STATA version 13 (College Station, Texas 77,845 USA) was used to perform meta-analysis of the proportion of *H. pylori* resistance rates to clarithromycin, metronidazole, amoxicillin and quinolones. In the analysis, a random-effects model was used to calculate the pooled (weighted) resistance rates for each antibiotic. Moreover, for percentages, the 95% confidence intervals were calculated. Heterogeneity was assessed by Cochran’s Q test(I^2^).

## Results

### Study selection

A total of 732 references were finally located through the search in academic databases and reduced to 389 after duplicate removal. In order to find grey materials, the search string was broaden and adapted to available search tools in the different sources. The grey search resulted in 78 references with a final number of 62 after de- duplication.

A total of 26 studies from different parts of Africa published between 1986 and 2017 were found and reviewed. There were six studies from Nigeria, three from South Africa and Egypt; Kenya, Senegal and Ethiopia had two studies each, while Tunisia, Malawi, Congo Brazzaville, Gambia, Uganda, Morocco, Algeria and Cameroon had one study each (Table 1). There were 4324 patients involved in the study. Most of the patients aged between 1 ½ and 90 yrs. These studies involved between 1 to 25 antibiotics among which some are not used in the *H.pylori* treatment regimens. The statistical heterogeneity was quantified and reported for each antibiotic resistance by I square. Overall studies included in this review were heterogeneous and the I square remained significant even after removing the outliers.

**Table 1:** Baseline characteristics of 26 studies in Africa from 1992 to 2017

Author	Country	Publication year	Sample size	Age Mean ± SD	Age Range	Drugs	Ref
Abdurashhed Abdul	Nigeria	2005	25	49.3 ± 16.8	20-73	Amo,Cip,Cla,clind,Eryth,Met,tet	[20]
Aboderin Oladiipo	Nigeria	2007	32	48.6 ± 16.23	20-73	Amo,Tet,Cip, Cla,Eryth,Met,,Rifamp	[21]
Adeniyi A.Bolande	Nigeria	2012	52	–	10-90	Aug,Amo,Tet,Eryth,Clox,Genta,Cotr,Chlor,Nitro,Nalid,Oflox,Cla,met,Cefs,Ceph,Vanc,Tob,Kana,Clind	[63]
Angol C Denish	Uganda	2017	142	40 ± 18	13-85	Cla,quino	[64]
Ani Agatha.E	Nigeria	1999	87	–	–	Amo,Cla,Met,Tet	[26]
Asrat Daniel	Ethiopia	2004	50	–	–	Cla,Eryth,Tet,Met,Amo	[24]
Bouihat Najat	Morocco	2016	255			Met, Cla, levo, Rifam, Amox, Tet	[33]
Fathi Marwa S	Egypt	2013	60	46.7 ± 8.8	50–60	Cla,Cip,Tet,Met,Amo	[22]
Ghaith Doaa	Egypt	2016	100	44 ± 11	21-60	Cla	[65]
Hadibi D.Fazia	Algeria	2016	195			Cla	[66]
Harries A.D	Malawi	1992	136	–	18-70	Met,Pen,Tet,Amo	[29]
Harrison Ute	Nigeria	2017	577			Met,Amox,Cla,Tet	[67]
Kimanga Andrew Nyerere	Kenya	2010	70	–	–	Amo,Met,Cla	[4]
Lwai-lume L	Kenya	2005	267	45.4 ± 17.6	15 - 85	Cla,Amo,Tet,Met	[68]
Mansour Khansa Ben	Tunisia	2010	273	38.3	–	Amo,Met,Cla	[27]
Nicoline F. Tanih	South Africa	2010	200	–	–	Amo,Cip,Cla,Gent,Eryth,Met,Tet	[31]
Nicoline F. Tanih	South Africa	2011	254	45.5 + 15.7	5-93	Met,Cla	[55]
Nicoline F. Tanih	South Africa	2013	254	–	–	Cla, Quin	[34]
Ngoyi, Ontsira Nina E	Congo Brazavil	2015	63	43.9 ± 15.3	17- 76	Cla,Tet,Quin,	[32]
Ndip N.Roland	Cameroon	2008	77	44.5 ± 15.7	15–77	Met,Tet,AMo,Cla	[69]
Seck Abdoulaye	Senegal	2009	40	–	–	Met, Cip,Amo	[28]
Seck Abdoulaye	Senegal	2013	108	45.3	18–93	Amo, Tet,Met, Cla, Lev	[30]
SeckaOusman	Gambia	2013	169	30	1 ½ -70	Met,Cla,Tet,Amo	[25]
Thor-H. Henriksen	Ethiopia	1999	290	–	–	Amo,Dox,Met	[18]
Sherif May	Egypt	2004	48	–	2-17	Amp,Cla,Eryth,Azyth,Met,Cip	[23]
Smith *I. Stella*	Nigeria	2001	532	–	–	Cip,Met,Amox,Pip,Ery,Imep,Gent,kan,ofl,Nor,Cef	[70]
Total			4324		1 ½-93		

Amo: Amoxicillin; Amp:Ampicillin; Aug: Augumentine:;Azyth: Azythromycin; Cip: Ciprofloxacin; Cla: Clarithromycin; Clin: Clindamycin; Clox: Cloxacicilin; Cotri:cotrimoxazol; Dox; Doxycycline; Cef:ceftriaxone; Ceph: Cephalexin; Eryth: Erythromycin; Gent:Gentamycin; Kan:Kanamycin; Met: Metronidazole; Nal:Nalidixic acid; Oflo:Ofloxacin; Tet: Tetracycline; Lev: Levofloxacin;Rifamp: RifampicinRifam: Rifamycin; Fur: Furazolidon; Quin:Quinolones; Van:Vancomycin

### Description of the studies

The review included 26 articles research articles, data in these articles were collected between 1986 and 2017. The majority of the studies were done in West Africa and East Africa. Out of 26 studies, 13(50%) used both disc method and E-test method for susceptibility testing, while 10 studies used PCR and culture, and 4 studies used only PCR technique (Table 2). The presence or absence of mutations conferring resistance to various antibiotics was reported in 18 articles and only one study did follow up of the patients to assess treatment outcome.

**Table 2:** Methods of identification and susceptibility method used

AUTHOR		METHOD USED		SUSCEPTIBILITY METHOD	INTERPRETATION CRITERIA	REF
	Isolate	CULTURE	PCR			
Abdurashhed et al	20	YES	NO	disc diffusion	NCCLS	[71]
Aboderin et al. 2007	31	YES	NO	disc diffusion	NCCLS	[71]
Adeniyi et al. 2012	33	YES	NO	disc diffusion	not documented	–
Angol C Denish	21	NO	YES	–	–	[64]
Ani et al. 1999	55	YES	NO	E-test	Thomsbbery, C. (1985);Hachem, C. Y., (1996).	[72, 73]
Asrat et al. 2004	50	YES	NO	E-test	Cederbrant G,1993	[74]
Bouihat Najat	255	YES	YES	E-test Disc diffusion	CLSI	[33]
Fathi et al... 2013	16	YES	YES	E-test, Disc diffusion	CLSI	[75]
Ghaith et al. 2016	70	NO	YES	–	–	[65]
Hadibi et al. 2016	91	NO	YES	–	–	[66]
Harries et al. 1992	50	YES	NO	disc diffusion	not documented	–
Harrison et al. 2016	111	YES	YES	E-Test	CLSI	[67]
Kimanga et al. 2000	65	YES	NO	E-test	CLSI	[75]
Lwai-Lume et al. 2005	108	YES	NO	E-test	NCCLS	–
Mansour et al. 2010	273	YES	YES	E-test	Chaabouni H, (2004)	[76]
Nicoline et al. 2010	191	YES	NO	disc diffusion	CLSI	[75]
Nicoline et al. 2011	200	YES	YES	disc diffusion	CLSI	[75]
Nicoline et al. 2013	78	YES	YES	disc diffusion	N. F. Tanih (2010)	[31]
Ngoyi et al. 2015	56	NO	YES	NA	NA	–
Roland et al. 2008	132	YES	YES	disc diffusion	NCCLS	[71]
Seck et al. 2009	40	YES	NO	E-test	Megraud F (1999)	[77]
Seck et al. 2013	108	YES	YES	E-test	CLSI	[75]
Secka et al. 2013	64	YES	YES	E-test	not documented	–
Sherif et al. 2004	48	YES	NO	E-test	not documented	–
Smith et al. 2001	245	YES	YES	E-test, Disc diffusion	NCCLS	[70]
Thor-Henric et al. 1999	19	YES	NO	disc diffusion	Cederbrant G,.1993	[74]
TOTAL	2430					

#### Susceptibility testing

**Fig. 2:**
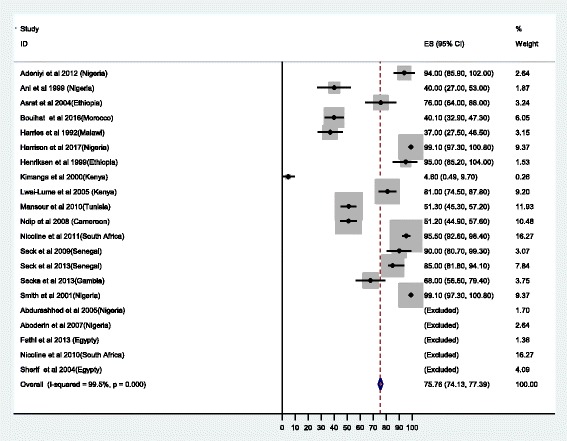
Proportional estimate of metronidazole with 95% confidence interval. Midpoint of each horizontal line segments shows the proportional estimate of metronidazole resistance for each study while the rhombic mark shows the pooled proportions for all studies

**Fig. 3:**
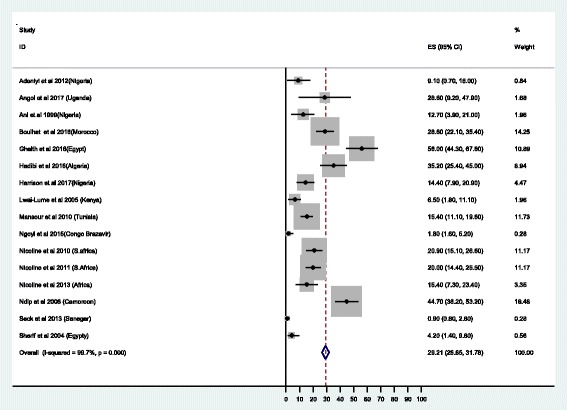
Proportional estimate of clarithromycin with 95% confidence interval. Midpoint of each horizontal line segments shows the proportional estimate of clarithromycin resistance for each study while the rhombic mark shows the pooled proportions for all studies

**Fig. 4:**
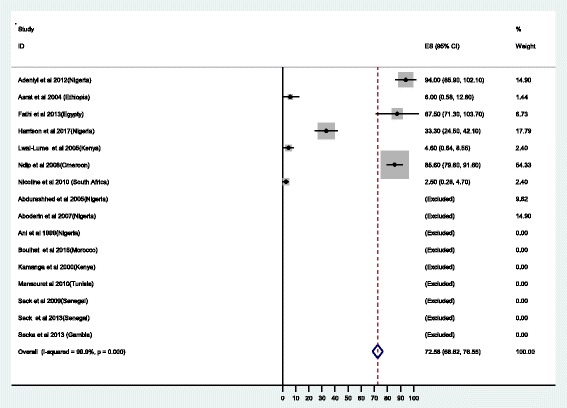
Proportional estimate of amoxicillin with 95% confidence interval. Midpoint of each horizontal line segments shows the proportional estimate of amoxicillin resistance for each study while the rhombic mark shows the pooled proportions for all studies

Despite high heterogeneity of the studies of more than 99%, the pooled susceptibility results have been reported with clear documentation of the number of isolates tested. A total of 2430 isolates were tested for various antibiotics using different methods as detailed in Table 2. Out of 2085 isolates tested for metronidazole, the resistance was found to range from 4.6% in the study in Kenya [19] to 100% in Egypt and Nigeria [20–23]; the overall resistance was 75.8% (95% CI: 74.1-.77.4) (Fig. 2). Regarding clarithromycin, the potent first line drug for *H. pylori* treatment the rate of resistance ranged from 0% in Gambia, Kenya and Ethiopia to 100% in Egypt and Nigeria [19–22, 24, 25], with overall resistance of 29.2%, (95%CI:26.7-31.8) (Fig. 3). Among 1530 isolates tested for amoxicillin, 72.6% (95% CI: 68.6-76.6) were found to be resistant (Fig. 4) [19–21, 26–28]. Of 1277 isolates tested for tetracycline, the overall resistance was 48.7%(95%CI: 44.5-52.9) [20, 24, 25, 29, 30]. Regarding quinolones, a total of 823 *H.pylori* isolates were tested, the resistance ranged from 0% to 32% [20, 28, 31, 32] with the overall resistance of 17.4% (95%CI 12.8 - 21.9). The resistance to rifampicin was 87.1% and none of the isolates were resistant to rifamycin [21, 33].

#### *H.pylori* mutations in Africa

A total of 334 isolates were tested for various mutations conferring resistance to clarithromycin. The commonest mutation detected was A2143G which was observed in 49 isolates (Table 3). Out of 60 isolates tested for quinolone mutations, 40(66.7%) were found to have mutations conferring resistance to quinolones. The commonest mutations was D87I which occurred in 16 isolates, followed by D91N occurring in 14 isolates [30, 32, 34]. The rdxA (41/66) was the commonest mutation detected to confer resistance to metronidazole [22, 25, 31]. Mutations conferring resistance to tetracycline were investigated in 40 isolates, and only one isolate was found to carry AGC926-928 mutation [32] (Table 3).

**Table 3:** Mutations distribution

DRUGS	Isolates tested for mutations	Total mutation detected	Type of Mutations	Frequency (%)
CLARITHROMYCIN	334	97	A2143G	49(50.5%)
			A2142G	24 (25%)
			A2147G	17(17.5%)
			A2146C	1 (1%)
			A2142C	2(2%)
			A2144G	2(2%)
			A2143C	2(2%)
QUINOLONES	60	40	D87I	16 (40%)
			D91N	14 (35%)
			D91G	1(2.5%)
			D91Y	1(2.5%)
			N87 K	8 (20%)
METRONIDAZOLE	66	56	RdxA (41)	41(73.2%)
			FrxA (15)	15(26.8%)
TETRACYCLINE	40	1	AGA926-928	1 (100%)

## Discussion

Treatment guidelines for the management of *H. pylori* infection in developing countries have recommended first, second and rescue therapy depending on the local susceptibility pattern [8, 9] however, this is not routinely practiced due to the poor resources in these regions. As a result of increased resistance to *H. pylori*, the eradication rates of *H.pylori* have been found to be lower than 80% [9–11].

The worldwide prevalence of metronidazole resistance has been found to range from is 31% - 53% in Europe and South America, and between 64% and 80% in Iran and Saudi Arabia [15, 35, 36]. In this review despite high heterogeneity of the studies the overall metronidazole resistance was 75.8% which is significantly higher than that observed in Europe and America. The use of metronidazole in the treatment of other endemic diseases such as diarrheal and protozoa diseases, could explain the significantly high rate of metronidazole resistance in Africa. It should be noted that the regimen used to treat other conditions provide sub-optimal concentration that cannot eradicate *H. pylori* strains hence selection of resistant *H.pylori* strains [37, 38].

In contrast to the observed high metronidazole resistance, the overall clarithromycin resistance in Africa was almost the same as that observed in North America (30.8%) and in Portuguese (42.3%). However, the observed resistance was significantly higher than that observed in middle Eastern countries (0-8%) [10, 36] and that documented in some parts of Europe and South America [15]. The use macrolides in outpatients setting has been associated with clarithromycin resistance [39]. In Africa, the resistance to clarithromycin could be linked to the high use of macrolides in the treatment of communicable diseases which are very prevalent, further studies to confirm this are warranted.

Regarding amoxicillin, the observed overall resistance was 72.6%, which is significantly higher than that observed in Europe (0.35%), North America (2%), South America (6.6%) and Asia (23.6%). Similar trend of the amoxicillin resistance has been observed in other pathogens [40]. In Africa amoxicillin/ampicillin is the most abused antibiotics both in rural and in urban areas [41] because it is cheaply available in oral formulation. Additionally, in Africa the observed tetracycline resistance (49.8%) was comparable to that in Asia which was found to range from 0.01% in Japan to 53.8% in India [15]. This high resistance to tetracycline could lead to the failure of novel three-in-one capsule of bismuth quadruple therapy that has proven effective as the first line of treatment in areas of high clarithromycin or metronidazole resistance [13]. It should be noted that this high resistance to tetracycline in Africa could be explained by the fact that tetracycline is readily misused in the treatment of pneumonia, acne and sexually transmitted infections [42] hence selecting for *H.pylori* resistant strains.

The rate of quinolone resistance observed in Africa is almost similar to that documented in South America (21%), Asia(25.3%) and North America (19%) but higher than that in Europe (14.2%) [15].

Rifabutin-based triple therapy has been found to be a useful salvage therapy in treatment of *H.pylori*. Rifabutin is derived from rifampicin and is used in rescue treatment of tuberculosis, this has also antimicrobial activity against *H. pylori*. In Africa there is no studies which were done on rifabutin instead two studies reported on rifamycin 87% in 2009 and rifampicin 0% in 2016 which shows the resistance is declining in this drug group. This might be due to the careful use of anti-Tuberculosis drugs.

### Mutations

*H, pylori* strains in Africa were found to carry A2143G, A2142G, A2147G and A2146C mutations that can lead to clarithromycin-resistance. Similar mutations have been observed in Asia [43, 44] and in South America, Europe and North America [45–49]. This can be due to migration process of human being [50]. In other part of the World in addition to these mutations other mutations in different positions have been found to confer clarithromycin resistance in *H pylori* strains (T2182C, T2190C, C2195T, A2223G, G2141A, C2694A, G2224A, C2245T, T2289C) [51–53]. The addition mutations in other part of the World can be due to phylogeographic tree differences of *H. pylori* by either gain(recombination) or gene loss(loss by deletion) in multiple strains which result in sequence and gene content diversity [54]. Metronidazole mutations [22, 25, 55] and quinolones mutations [56–58] observed in Africa were similar to that observed in Europe, in Asia and America.

In our review *H.pylori* mutation conferring resistance to tetracycline had two base pair mutation AGC926-928 as observed in Congo Brazzaville, similar mutation was obtained in other parts of the world, in America [59, 60], Europe and Asia [61, 62].

One of the major limitation of this review is significantly high heterogeneity of the studies, this could be due to lack of methods standardization, heterogeneity of the population, and difference in discs quality. Despite this limitation the magnitude the data have been presented to highlight the importance of the problem and the need for standardized surveillance system.

## Conclusion

Significantly high proportion of *H. pylori* strains in Africa are resistant to metronidazole, clarithromycin and amoxicillin. There is a need of conducting standardized surveillance of *H.pylori* susceptibility pattern in Africa to provide data that can be used to establish effective empirical treatment.

## Additional files

Additional file 1: African countries included in the systematic search based on PubMed’s. (DOCX 25 kb)

Additional file 2: Academic Databases. (DOCX 26 kb)

## Abbreviations

CLSI: The Clinical Laboratory Standards Institute
E-Test: Epsilometer test
FrxA: gene which encodes for NAD (P) H Flavin oxidoreductase
NA: Not applicable
NCCLS: National Committee for Clinical Laboratory Standards (currently is known as Clinical laboratory Standard Institute (CLSI)
PCR: Polymerase chain reaction
RdxA: gene, which encodes an oxygen- insensitive NADPH Nitroreductase
