# Supplementary material for: The magnitude of antibiotic resistance to Helicobacter pylori in Africa and identified mutations which confer resistance to antibiotics: systematic review and meta-analysis
Source: BMC Infect Dis. 2018 Apr 24;18:193. doi: 10.1186/s12879-018-3099-4 (PMC5921563; doi:10.1186/s12879-018-3099-4)
Supplement: Supplementary file 1 — African countries included in the systematic search based on PubMed’s. (DOCX 25 kb) [file 12879_2018_3099_MOESM1_ESM.docx]

**Additional File 1**

**African countries included in**

**the systematic search based on PubMed’s MeSH**

All African countries were identified with the help of PubMed’s MeSH. A list of all previous country names was furthermore compiled based on MeSH **“**Entry Terms” for each country, and reviewed to only included names from 1986- (here marked with “Included” or “Searched by…” in the notes field).

| **Country** | **Previous country names** | **Notes** |
| --- | --- | --- |
| **Algeria** | N/A |  |
| **Angola** | N/A |  |
| **Benin** | a. Dahomey  b. “Republic of Benin” | a. Not relevant  b. Searched by “Benin” |
| **Botswana** | a. Bechuanaland  b. Kalahari | a. Not relevant  b. Not relevant |
| **Burkina Faso** | a. Upper Volta  b. Burkina Fasso | a. Included  b. Included |
| **Burundi** | a. Republic of Burundi  b. Urundi | a. Searched by “Burundi”  b. Not relevant |
| **Cameroon** | a. Republic of Cameron  b. United Republic of Cameroon  c. Cameroons | a. Included  b. Searched by “Cameroon”  c. Not relevant |
| **Cape Verde** | Republic of Cape Verde | Searched by “Cap Verde” |
| **Central African Republic**  (Searched by “Africa*”) | Ubangi-Shari | Not relevant |
| **Chad** | N/A |  |
| **Congo** | a. Republic of the Congo  b. Congo (Brazzaville) | a. Searched by “Congo”  b. Searched by “Congo” |
| **Côte d'Ivoire** | a. Ivory Coast  b. Republic of Côte d'Ivoire | a. Included  b. Searched by “Côte d'Ivoire” |
| **Djibouti** | a. Somaliland, French  b. Republic of Djibouti  c. French Somaliland | a. Not relevant  b. Searched by “Djibuti”  c. Not relevant |
| **Democratic republic of Congo**  (Searched by “Congo”) | a. Congo (Kinshasa)  b. Zaire  c. Belgian Congo  d. Katanga | a. Searched by “Congo”  b. Included  c. Searched by “Congo”  d. Not relevant |
| **Egypt** | 1. a. Arab Republic of Egypt 2. b. United Arab Republic | a. Searched by “Egypt”  b. Not relevant |
| **Equatorial Guinea**  (Searched by “Guinea”) | a. Republic of Equatorial Guinea  b. Spanish Guinea  c. Guinea, Spanish  d. Rio Muni | a. Searched by “Equatorial Guinea”  b. Not relevant  c. Not relevant  d. Not relevant |
| **Eritrea** | N/A |  |
| **Ethiopia** | Federal Democratic Republic of Ethiopia | Searched by “Ethiopia” |
| **Gabon** | Gabonese Republic | Searched by “Gabon*” |
| **Gambia** | Republic of the Gambia | Searched by “Gambia” |
| **Ghana** | a. Republic of Ghana  b. Gold Cost | a. Searched by “Ghana”  b. Not relevant |
| **Guinea** | a. Guinea, French  b. Republic of Guinea  c. French Guinea  d. Guinea, Republic of | a. Searched by “Guinea”  b. Searched by “Guinea”  c. Searched by “Guinea”  d. Searched by “Guinea” |
| **Guinea-Bissau** | a. Republic of Guinea-Bissau  b. Portuguese Guinea  c. Guinea, Portuguese  d. Guinea-Bissau, Republic of | a. Searched by “Guinea-Bissau”  b. Searched by “Guinea”  c. Searched by “Guinea”  d. Searched by “Guinea-Bissau” |
| **Kenya** | Republic of Kenya | Searched by “Kenya” |
| **Lesotho** | a. Basutoland  b. Kingdom of Lesotho | a. Not relevant  b. Searched by “Lesotho” |
| **Liberia** | Republic of Liberia | Searched by “Liberia” |
| [**Libya**](https://www.ncbi.nlm.nih.gov/mesh/68008002) | N/A |  |
| **Malawi** | a. Republic of Malawi  b. Nyasaland | a. Searched by “Malawi”  b. Not relevant |
| **Mali** | Republic of Mali | Searched by “Mali” |
| **Mauritania** | N/A |  |
| [**Morocco**](https://www.ncbi.nlm.nih.gov/mesh/68009018) | Ifni | Not relevant |
| **Mozambique** | a. Republic of Mozambique  b. Portuguese East Africa | a. Searched by “Mozambique”  b. Searched by “Africa” |
| **Namibia** | a. Southwest Africa  b. Republic of Namibia  c. South West Africa | a. Searched by “Africa”  b. Searched by “Namibia”  c. Searched by “Africa” |
| **Niger** | Republic of Niger | Searched by “Niger” |
| **Nigeria** | Federal Republic of Nigeria | Searched by “Nigeria” |
| **Rwanda** | a. Republic of Rwanda  b. Ruanda | a. Searched by “Rwanda”  b. Not relevant |
| **Senegal** | Republic of Senegal | Searched by “Senegal” |
| **Sierra Leone** | Republic of Sierra Leone | Searched by “Sierra Leone” |
| **Somalia** | N/A |  |
| **South Africa**  (Searched by “Africa*”) | a. Union of South Africa  b. Republic of South Africa | a. Searched by “Africa”  b. Searched by “Africa” |
| **South Sudan**  (Searched by “Sudan”) | N/A |  |
| **Sudan** | Republic of the Sudan | Searched by “Sudan” |
| **Swaziland** | N/A |  |
| **Tanzania** | a. United Republic of Tanzania  b. Zanzibar  c. Tanganyika | a. Searched by “Tanzania”  b. Not relevant  c. Not relevant |
| **Togo** | Togolese Republic | Included |
| **Tunisia** | N/A |  |
| **Uganda** | Republic of Uganda | Searched by “Uganda” |
| **Zambia** | a. Rhodesia, Northern  b. Northern Rhodesia  c. Republic of Zambia | a. Not relevant  b. Not relevant  c. Searched by “Zambia” |
| **Zimbabwe** | a. Zimbabwe Rhodesia  b. Southern Rhodesia  c. Republic of Zimbabwe  d. Rhodesia, Southern | a. Searched by “Zimbabwe”  b. Searched by “Zimbabwe”  c. Searched by “Zimbabwe”  d. Not relevant |
