# Supplementary material for: The magnitude of antibiotic resistance to Helicobacter pylori in Africa and identified mutations which confer resistance to antibiotics: systematic review and meta-analysis
Source: BMC Infect Dis. 2018 Apr 24;18:193. doi: 10.1186/s12879-018-3099-4 (PMC5921563; doi:10.1186/s12879-018-3099-4)
Supplement: Supplementary file 2 — Academic Databases. (DOCX 26 kb) [file 12879_2018_3099_MOESM2_ESM.docx]

**Additional File 2**

**Literature search**

**Academic Databases**

| **Database** | **Search String** | **Result** | **Notes** |
| --- | --- | --- | --- |
| **PubMed**  **Coverage:** 1809-  **Search date:** June 1^th^, 2017 | (((("Ampicillin Resistance"[Mesh] OR "Penicillin Resistance"[Mesh] OR "Tetracycline Resistance"[Mesh] OR "Drug Resistance, Bacterial"[Mesh] OR "Drug Resistance, Multiple, Bacterial"[Mesh] OR Resistan*[Title/Abstract]))) AND (("H pylori"[Title/Abstract] OR "Helicobacter pylori"[Title/Abstract] OR "Helicobacter pylori"[Mesh] OR "Helicobacter Infections"[Title/Abstract] OR "Helicobacter Infection"[Title/Abstract] OR "Helicobacter Infections"[Mesh] OR"Campylobacter"[Mesh] OR Campylobacter [Title/Abstract]) AND (Rifabutin[Title/Abstract] OR Antibiotic*[Title/Abstract] OR Tinidazole[Title/Abstract] OR Doxycycline[Title/Abstract] OR Ampicillin[Title/Abstract] OR Moxifloxacin[Title/Abstract] OR Metronidazole[Title/Abstract] OR Clarithromycin[Title/Abstract] OR Levofloxacin[Title/Abstract] OR Amoxicillin[Title/Abstract] OR “combination capsules”[Title/Abstract] OR “combination capsule”[Title/Abstract] OR “Sequential therapy”[Title/Abstract] OR "Quad therapy”[Title/Abstract] OR “Quadruple therapy"[Title/Abstract] OR “Triple therapy”[Title/Abstract] OR Penicillin*[Title/Abstract] OR Macrolide*[Title/Abstract] OR Rifamycin*[Title/Abstract] OR Nitroimidazole*[Title/Abstract] OR Quinolone*[Title/Abstract] OR Tetracycline*[Title/Abstract] OR Quinolinone*[Title/Abstract] OR "anti-bacterial agents"[Title/Abstract] OR "anti-bacterial agent"[Title/Abstract] OR amoxycillin[Title/Abstract] OR "Anti-Bacterial Agents"[Mesh] OR "Penicillins"[Mesh] OR "Macrolides"[Mesh] OR "Rifamycins"[Mesh] OR "Nitroimidazoles"[Mesh] OR "Quinolones"[Mesh] OR "Tetracyclines"[Mesh]))) AND (((("Republic of Cameron"[Title/Abstract] OR "Africa"[Mesh] OR “Sub Sahara”[Title/Abstract] OR “Sub Saharan”[Title/Abstract] OR Angola [Title/Abstract] OR Benin [Title/Abstract] OR Botswana [Title/Abstract] OR “Burkina Faso”[Title/Abstract] OR Burundi [Title/Abstract] OR Cameroon[Title/Abstract] OR “Cape Verde”[Title/Abstract] OR Chad[Title/Abstract] OR Congo[Title/Abstract] OR “Côte d'Ivoire”[Title/Abstract] OR “Ivory Coast”[Title/Abstract] OR Djibouti[Title/Abstract] OR “Equatorial Guinea”[Title/Abstract] OR Eritrea[Title/Abstract] OR Ethiopia[Title/Abstract] OR Gabon*[Title/Abstract] OR Gambia[Title/Abstract] OR Ghana[Title/Abstract] OR Guinea[Title/Abstract] OR “Guinea Bissau”[Title/Abstract] OR Kenya[Title/Abstract] OR Lesotho[Title/Abstract] OR Liberia[Title/Abstract] OR Malawi[Title/Abstract] OR Mali[Title/Abstract] OR Mauritania[Title/Abstract] OR Mozambique[Title/Abstract] OR Namibia[Title/Abstract] OR Niger[Title/Abstract] OR Nigeria[Title/Abstract] OR Rwanda[Title/Abstract] OR Senegal[Title/Abstract] OR “Sierra Leone”[Title/Abstract] OR Somalia[Title/Abstract] OR Sudan [Title/Abstract] OR Swaziland[Title/Abstract] OR Tanzania[Title/Abstract] OR Togo[Title/Abstract] OR Uganda[Title/Abstract] OR Zambia[Title/Abstract] OR Zimbabwe[Title/Abstract] OR Africa*[Title/Abstract] OR "Togolese Republic"[Title/Abstract] OR "Burkina Fasso"[Title/Abstract] OR "Upper Volta"[Title/Abstract] OR Zaire[Title/Abstract]))) OR ((Algeria[Title/Abstract] OR Egypt[Title/Abstract] OR Libya[Title/Abstract] OR Morocco[Title/Abstract] OR Tunisia[Title/Abstract]))) | **131** | All terms searched in the field “Title/Abstract” and in “MeSH” when available.  No filters or limitations applied |
| **EMBASE**  (OVID)  **Coverage:** 1980-  **Search date:** June 11^th^, 2017 | (([Penicillin Resistance](http://0-ovidsp.tx.ovid.com.elibrary.qatar-weill.cornell.edu/sp-3.17.0a/ovidweb.cgi?&Controlled+Vocabulary=Mapping%7c0&Return=mapping&S=NMGFFPNIELDDCJBGNCJKJEJCOKDBAA00)/de OR Antibiotic Resistance/de OR  Multidrug Resistance/de OR Resistan*[Title/Abstract]) AND ("H pylori"[Title/Abstract] OR "Helicobacter pylori"[Title/Abstract] OR Helicobacter pylori/de OR "Helicobacter Infections"[Title/Abstract] OR "Helicobacter Infection"[Title/Abstract] OR Helicobacter Infections/de OR Campylobacter [Title/Abstract] OR Campylobacter/de) AND (Rifabutin[Title/Abstract] OR Antibiotic*[Title/Abstract] OR Tinidazole[Title/Abstract] OR Doxycycline[Title/Abstract] OR Ampicillin[Title/Abstract] OR Moxifloxacin[Title/Abstract] OR Metronidazole[Title/Abstract] OR Clarithromycin[Title/Abstract] OR Levofloxacin[Title/Abstract] OR Amoxicillin[Title/Abstract] OR “combination capsules”[Title/Abstract] OR “combination capsule”[Title/Abstract] OR “Sequential therapy”[Title/Abstract] OR "Quad therapy”[Title/Abstract] OR “Quadruple therapy"[Title/Abstract] OR “Triple therapy”[Title/Abstract] OR Penicillin*[Title/Abstract] OR Macrolide*[Title/Abstract]) OR Rifamycin*[Title/Abstract] OR Nitroimidazole*[Title/Abstract] OR Quinolone*[Title/Abstract] OR Tetracycline*[Title/Abstract] OR Quinolinone*[Title/Abstract] OR "anti-bacterial agents"[Title/Abstract]OR "anti-bacterial agent"[Title/Abstract] OR amoxycillin[Title/Abstract] OR Antibiotic Agent/de OR Penicillin Derivative/de OR Macrolide/de OR Rifamycin/ OR Nitroimidazole Derivative/de OR Quinolone Derivative/de OR Tetracycline Derivative/de) AND (Africa/exp OR “Sub Sahara”[Title/Abstract] OR “Sub Saharan”[Title/Abstract] OR Angola [Title/Abstract] OR Benin [Title/Abstract] OR Botswana [Title/Abstract] OR “Burkina Faso”[Title/Abstract] OR Burundi [Title/Abstract] OR Cameroon[Title/Abstract] OR "Republic of Cameron"[Title/Abstract] OR “Cape Verde”[Title/Abstract] OR Chad[Title/Abstract] OR Congo[Title/Abstract] OR “Cote d'Ivoire”[Title/Abstract] OR “Ivory Coast”[Title/Abstract] OR Djibouti[Title/Abstract] OR “Equatorial Guinea”[Title/Abstract] OR Eritrea[Title/Abstract] OR Ethiopia[Title/Abstract] OR Gabon*[Title/Abstract] OR Gambia[Title/Abstract] OR Ghana[Title/Abstract] OR Guinea[Title/Abstract] OR “Guinea Bissau”[Title/Abstract] OR Kenya[Title/Abstract] OR Lesotho[Title/Abstract] OR Liberia[Title/Abstract] OR Malawi[Title/Abstract] OR Mali[Title/Abstract] OR Mauritania[Title/Abstract] OR Mozambique[Title/Abstract] OR Namibia[Title/Abstract] OR Niger[Title/Abstract] OR Nigeria[Title/Abstract] OR Rwanda[Title/Abstract] OR Senegal[Title/Abstract] OR “Sierra Leone”[Title/Abstract] OR Somalia[Title/Abstract] OR Sudan [Title/Abstract] OR Swaziland[Title/Abstract] OR Tanzania[Title/Abstract] OR Togo[Title/Abstract] OR Uganda[Title/Abstract] OR Zambia[Title/Abstract] OR Zimbabwe[Title/Abstract]))) OR Africa*[Title/Abstract] OR "Togolese Republic"[Title/Abstract] OR Burkina Fasso [Title/Abstract] OR Upper Volta[Title/Abstract] OR Zaire[Title/Abstract] OR Algeria[Title/Abstract] OR Egypt[Title/Abstract] OR Libya[Title/Abstract] OR Morocco[Title/Abstract] OR Tunisia[Title/Abstract])) | **188** | All terms searched in the fields “Title” and “Abstract” (here marked with “[Title/Abstract]” after the term, and in “Thesaurus” (here marked with “/de” or “/exp” after the term) when available.  No filters or limitations applied  Non- English signs like ô does not responding, can’t be searched in this database and have therefor been replaces with English characters. i.e. “o” instead of “ô”. |
| **Web of Science** (all databases)  **Coverage:** 1864-  **Search date:** June 1^th^, 2017 | ((Resistan*) AND ("H pylori" OR "Helicobacter pylori" OR "Helicobacter Infections" OR "Helicobacter Infection" OR Campylobacter) AND (Rifabutin OR Antibiotic* OR Tinidazole OR Doxycycline OR Ampicillin OR Moxifloxacin OR Metronidazole OR Clarithromycin OR Levofloxacin OR Amoxicillin OR “combination capsules” OR “combination capsule” OR “Sequential therapy” OR "Quad therapy” OR “Quadruple therapy" OR “Triple therapy” OR Penicillin* OR Macrolide* OR Rifamycin* OR Nitroimidazole* OR Quinolone* OR Tetracycline* OR Quinolinone* OR "anti-bacterial agents" OR "anti-bacterial agent" OR amoxicillin) AND (“Sub Sahara” OR “Sub Saharan” OR Angola OR Benin OR Botswana OR “Burkina Faso” OR Burundi OR Cameroon OR "Republic of Cameron" OR “Cape Verde” OR Chad OR Congo OR “Côte d'Ivoire” OR “Ivory Coast” OR Djibouti OR “Equatorial Guinea” OR Eritrea OR Ethiopia OR Gabon* OR Gambia OR Ghana OR Guinea OR “Guinea Bissau” OR Kenya OR Lesotho OR Liberia OR Malawi OR Mali OR Mauritania OR Mozambique OR Namibia OR Niger OR Nigeria OR Rwanda OR Senegal OR “Sierra Leone” OR Somalia OR Sudan OR Swaziland OR Tanzania OR Togo OR Uganda OR Zambia OR Zimbabwe OR Africa* OR "Togolese Republic" OR "Burkina Fasso" OR "Upper Volta" OR Zaire OR Algeria OR Egypt OR Libya OR Morocco OR Tunisia)) | **272** | All terms searched in the field “Topic” (which includes “Abstract”, “Title”, Author Keywords and “Keyword Plus”)  Title and abstract search only is not available in this database.  No thesaurus available.  No filters or limitations applied |
| **Africa –Wide Information**  **Coverage:**  1825-  **Search date:** June 7^th^, 2017 | ((Resistan*) AND ("H pylori" OR "Helicobacter pylori" OR "Helicobacter Infections" OR "Helicobacter Infection" OR Campylobacter) AND (Rifabutin OR Antibiotic* OR Tinidazole OR Doxycycline OR Ampicillin OR Moxifloxacin OR Metronidazole OR Clarithromycin OR Levofloxacin OR Amoxicillin OR “combination capsules” OR “combination capsule” OR “Sequential therapy” OR "Quad therapy” OR “Quadruple therapy" OR “Triple therapy” OR Penicillin* OR Macrolide* OR Rifamycin* OR Nitroimidazole* OR Quinolone* OR Tetracycline* OR Quinolinone* OR "anti-bacterial agents" OR "anti-bacterial agent" OR amoxicillin) AND (“Sub Sahara” OR “Sub Saharan” OR Angola OR Benin OR Botswana OR “Burkina Faso” OR Burundi OR Cameroon OR "Republic of Cameron" OR “Cape Verde” OR Chad OR Congo OR “Côte d'Ivoire” OR “Ivory Coast” OR Djibouti OR “Equatorial Guinea” OR Eritrea OR Ethiopia OR Gabon* OR Gambia OR Ghana OR Guinea OR “Guinea Bissau” OR Kenya OR Lesotho OR Liberia OR Malawi OR Mali OR Mauritania OR Mozambique OR Namibia OR Niger OR Nigeria OR Rwanda OR Senegal OR “Sierra Leone” OR Somalia OR Sudan OR Swaziland OR Tanzania OR Togo OR Uganda OR Zambia OR Zimbabwe OR Africa* OR "Togolese Republic" OR "Burkina Fasso" OR "Upper Volta" OR Zaire OR Algeria OR Egypt OR Libya OR Morocco OR Tunisia)) | **141** | All terms searched in the fields “Title” and “Abstract”.  No thesaurus available  No filters or limitations applied |
| **Total numbers of references** | | | **732** |
| **Total numbers of references after de-duplication** | | | **389** |

**Grey Sources**

| **Source** | **Search string** | **Result** | **Notes** |
| --- | --- | --- | --- |
| **ProQuest Dissertation and Theses**  **Search date:**  June 1^th^ 2017 | ((Resistan*) AND ("H pylori" OR "Helicobacter pylori" OR "Helicobacter Infections" OR "Helicobacter Infection" OR Campylobacter) AND (Rifabutin OR Antibiotic* OR Tinidazole OR Doxycycline OR Ampicillin OR Moxifloxacin OR Metronidazole OR Clarithromycin OR Levofloxacin OR Amoxicillin OR “combination capsules” OR “combination capsule” OR “Sequential therapy” OR "Quad therapy” OR “Quadruple therapy" OR “Triple therapy” OR Penicillin* OR Macrolide* OR Rifamycin* OR Nitroimidazole* OR Quinolone* OR Tetracycline* OR Quinolinone* OR "anti-bacterial agents" OR "anti-bacterial agent" OR amoxicillin) AND (“Sub Sahara” OR “Sub Saharan” OR Angola OR Benin OR Botswana OR “Burkina Faso” OR Burundi OR Cameroon OR "Republic of Cameron" OR “Cape Verde” OR Chad OR Congo OR “Côte d'Ivoire” OR “Ivory Coast” OR Djibouti OR “Equatorial Guinea” OR Eritrea OR Ethiopia OR Gabon* OR Gambia OR Ghana OR Guinea OR “Guinea Bissau” OR Kenya OR Lesotho OR Liberia OR Malawi OR Mali OR Mauritania OR Mozambique OR Namibia OR Niger OR Nigeria OR Rwanda OR Senegal OR “Sierra Leone” OR Somalia OR Sudan OR Swaziland OR Tanzania OR Togo OR Uganda OR Zambia OR Zimbabwe OR Africa* OR "Togolese Republic" OR "Burkina Fasso" OR "Upper Volta" OR Zaire OR Algeria OR Egypt OR Libya OR Morocco OR Tunisia)) | **4** | All terms searched in “All fields except full text”.  No filters or limitations applied |
| **Ethos**  **Search date:**  June 1^th^ 2017 | "Helicobacter Pylori" AND Antibiotics | **10** | No search fields, filters or limitations applied.  The search functions in this source are very limited. A broad search was therefore conducted. |
| **Scopus**  **Search date:**  June 1^th^ 2017 | ((Resistan*) AND ("H pylori" OR "Helicobacter pylori" OR "Helicobacter Infections" OR "Helicobacter Infection" OR Campylobacter) AND (Rifabutin OR Antibiotic* OR Tinidazole OR Doxycycline OR Ampicillin OR Moxifloxacin OR Metronidazole OR Clarithromycin OR Levofloxacin OR Amoxicillin OR “combination capsules” OR “combination capsule” OR “Sequential therapy” OR "Quad therapy” OR “Quadruple therapy" OR “Triple therapy” OR Penicillin* OR Macrolide* OR Rifamycin* OR Nitroimidazole* OR Quinolone* OR Tetracycline* OR Quinolinone* OR "anti-bacterial agents" OR "anti-bacterial agent" OR amoxicillin) AND (“Sub Sahara” OR “Sub Saharan” OR Angola OR Benin OR Botswana OR “Burkina Faso” OR Burundi OR Cameroon OR "Republic of Cameron" OR “Cape Verde” OR Chad OR Congo OR “Côte d'Ivoire” OR “Ivory Coast” OR Djibouti OR “Equatorial Guinea” OR Eritrea OR Ethiopia OR Gabon* OR Gambia OR Ghana OR Guinea OR “Guinea Bissau” OR Kenya OR Lesotho OR Liberia OR Malawi OR Mali OR Mauritania OR Mozambique OR Namibia OR Niger OR Nigeria OR Rwanda OR Senegal OR “Sierra Leone” OR Somalia OR Sudan OR Swaziland OR Tanzania OR Togo OR Uganda OR Zambia OR Zimbabwe OR Africa* OR "Togolese Republic" OR "Burkina Fasso" OR "Upper Volta" OR Zaire OR Algeria OR Egypt OR Libya OR Morocco OR Tunisia)) | **1** | All terms in the fields “Article Title”, “Abstract” and “Keywords”  Filter applied: “Conference Papers” |
| **Africa Index Medicus (WHO)**  **Search date:**  June 1^th^ 2017 | "Helicobacter Pylori" | **38** | No search fields, filters or limitations applied.  The search functions in this source are very limited. A broad search was therefore conducted. |
| **Open Grey**  **Search date:**  June 1^th^ 2017 | "Helicobacter pylori" AND Africa | **2** | No search fields, filters or limitations applied.  The search functions in this source are very limited. A broad search was therefore conducted. |
|  | "Helicobacter Pylori" AND Antibiotics | **8** | No search fields, filters or limitations applied. |
| **BASE**  **Search date:**  June 1^th^ 2017 | "Helicobacter Pylori" AND "Antibiotics Resistance" AND Africa | **19** | No search fields, filters or limitations applied.  The search functions in this source are very limited. A broad search was therefore conducted. |
| **Total numbers of references** | | | **82** |
| **Total numbers of references after de-duplication** | | | **62** |
